# Dyscalculia and dyslexia: Different behavioral, yet similar brain activity profiles during arithmetic

**DOI:** 10.1016/j.nicl.2018.03.003

**Published:** 2018-03-04

**Authors:** Lien Peters, Jessica Bulthé, Nicky Daniels, Hans Op de Beeck, Bert De Smedt

**Affiliations:** a Parenting and Special Education Research Unit, KU Leuven, Belgium; b Brain and Cognition Research Unit, KU Leuven, Belgium; c Numerical Cognition Laboratory, Department of Psychology, Brain and Mind Institute, Western University, Canada

**Keywords:** Arithmetic, Children, Dyscalculia, Dyslexia, fMRI

## Abstract

Brain disorders are often investigated in isolation, but very different conclusions might be reached when studies directly contrast multiple disorders. Here, we illustrate this in the context of specific learning disorders, such as dyscalculia and dyslexia. While children with dyscalculia show deficits in arithmetic, children with dyslexia present with reading difficulties. Furthermore, the comorbidity between dyslexia and dyscalculia is surprisingly high. Different hypotheses have been proposed on the origin of these disorders (number processing deficits in dyscalculia, phonological deficits in dyslexia) but these have never been directly contrasted in one brain imaging study. Therefore, we compared the brain activity of children with dyslexia, children with dyscalculia, children with comorbid dyslexia/dyscalculia and healthy controls during arithmetic in a design that allowed us to disentangle various processes that might be associated with the specific or common neural origins of these learning disorders.

Participants were 62 children aged 9 to 12, 39 of whom had been clinically diagnosed with a specific learning disorder (dyscalculia and/or dyslexia). All children underwent fMRI scanning while performing an arithmetic task in different formats (dot arrays, digits and number words). At the behavioral level, children with dyscalculia showed lower accuracy when subtracting dot arrays, and all children with learning disorders were slower in responding compared to typically developing children (especially in symbolic formats). However, at the neural level, analyses pointed towards substantial neural similarity between children with learning disorders: Control children demonstrated higher activation levels in frontal and parietal areas than the three groups of children with learning disorders, regardless of the disorder. A direct comparison between the groups of children with learning disorders revealed similar levels of neural activation throughout the brain across these groups. Multivariate subject generalization analyses were used to statistically test the degree of similarity, and confirmed that the neural activation patterns of children with dyslexia, dyscalculia and dyslexia/dyscalculia were highly similar in how they deviated from neural activation patterns in control children. Collectively, these results suggest that, despite differences at the behavioral level, the brain activity profiles of children with different learning disorders during arithmetic may be more similar than initially thought.

## Highlights

• We investigated the neural correlates of arithmetic in children with dyslexia and/or dyscalculia and controls.
• Univariate and multivariate analyses pointed towards substantial neural similarity between children with learning disorders.
• Despite behavioral differences, brain activity profiles of children with learning disorders were more similar than expected.

## 1 Introduction

Neurodevelopmental disorders, such as specific learning disorders, ADHD and autism, are consistently investigated in isolation, leaving direct comparisons of the neurobiological origins of different neurodevelopmental disorders and their specificity uninvestigated to date. In the current study, we focused on specific learning disorders, such as difficulties in learning to calculate (dyscalculia) or to read (dyslexia), which are very common and affect between 5 and 15% of primary school children (Gaddes, 2013; Peterson and Pennington, 2015; Rapin, 2016). The prevalence of the combination of both, comorbid dyslexia/dyscalculia, is very high (Dirks et al., 2008), yet to date there has been no neuroimaging research performed investigating the neurobiological origin of this comorbidity. Even more, this high comorbidity has been vastly overlooked in previous neuroimaging research in these disorders, as arithmetic ability is often not taken into account in dyslexia research, and children with low reading ability are typically excluded from dyscalculia studies. In this study, we therefore directly compared the neural profiles of children with dyscalculia and/or dyslexia, allowing us for the first time to investigate the specificity or commonality of the neural origin of these learning disorders.

Specific learning disorders have been found to be associated with higher rates of high school dropout, higher levels of psychological distress, higher rates of unemployment and lower income in later life (American Psychiatric Association, 2013). Research has thus far mainly focused on differentiating the cognitive deficits associated with these specific learning disorders: deficits in number processing for dyscalculia (Ansari, 2008; Ashkenazi et al., 2013; Butterworth et al., 2011; Mazzocco et al., 2011; Rousselle and Noël, 2007), and deficits in phonological processing for dyslexia (Gabrieli, 2009; Ozernov-Palchik et al., 2016; Stanovich et al., 1994; Wagner and Torgesen, 1987). On the other hand, it turns out that difficulties in arithmetic, which are obviously the hallmark of dyscalculia, are also remarkably common in dyslexia, particularly when it comes to retrieving arithmetic facts from semantic long-term memory, as is the case in multiplication (De Smedt and Boets, 2010; Göbel, 2015; Simmons and Singleton, 2008; Träff and Passolunghi, 2015). A possible explanation for this finding is that arithmetic fact retrieval might be influenced by phonological processes (De Smedt et al., 2010; Dehaene et al., 2003; Geary and Hoard, 2001), which are presumed to be the key cognitive deficits in children with dyslexia. Given these shared deficits in arithmetic in both dyscalculia and dyslexia, we opted to use an arithmetic task in the context of this study.

Turning to the origin of the comorbidity between dyslexia and dyscalculia, most studies have reported additive effects, as children with comorbid dyslexia/dyscalculia showed similar deficits compared to children with dyslexia in reading, and similar deficits compared to children with dyscalculia in arithmetic (Landerl et al., 2009; Kristina Moll et al., 2015). Studies that have investigated the possibility of domain-general factors contributing to the comorbidity, have reported that, for example, working memory and naming speed are also implicated in comorbid dyslexia/dyscalculia (Maehler and Schuchardt, 2016; Moll et al., 2016; Willburger et al., 2008; Willcutt et al., 2013; Wilson et al., 2015). Collectively, the body of work investigating the comorbidity between learning disorders is small to date and more studies are necessary to gain more insight into what underlies this comorbidity.

The nascent body of developmental brain imaging studies has indicated that arithmetic recruits a network of various brain regions and that this network involves the integrity of several white matter pathways (see Menon, 2015; Peters and De Smedt, 2017). This arithmetic network comprises inferior and posterior parietal areas, as well as temporoparietal regions (e.g., supramarginal and angular gyri), the fusiform gyrus, hippocampus and prefrontal regions. Research has consistently reported a frontal-to-parietal shift with development: As children gain more experience with arithmetic, they show a decrease in activation in the prefrontal areas, yet an increase in reliance on parietal areas (Peters and De Smedt, 2017). Furthermore, behavioral research in children has revealed a shift in the strategies children use to solve arithmetic problems, from reliance on procedural strategies towards retrieving solutions from long-term memory (Ashcraft, 1982; Geary et al., 1987). This behavioral finding is supported by findings at the neural level, indicated by a shift from more engagement of the intraparietal sulci and prefrontal cortex towards increased reliance on memory-related, temporoparietal (e.g., supramarginal and angular gyri) and hippocampal regions (Menon, 2015; Peters and De Smedt, 2017).

The limited amount of neuroimaging research in children with *dyscalculia* has so far shown mixed results of both hypo- (i.e., less activation) and hyper-activation (i.e., more activation) in this whole brain network in children with dyscalculia compared to their typically developing peers (Ashkenazi et al., 2012; Berteletti et al., 2014; Davis et al., 2009; De Smedt et al., 2011; Rosenberg-Lee et al., 2015). The existing body of evidence thus remains unclear in how the arithmetic network is recruited in children with dyscalculia. These inconsistent findings could be attributed to study differences in terms of paradigms and control tasks used (i.e., addition vs. multiplication vs. approximate arithmetic), analysis approach (i.e., region of interest vs. whole brain analyses), age group, and the cut-off criteria used to define dyscalculia.

Only one study to date has investigated the neural correlates of arithmetic in children with *dyslexia*. Evans et al. (2014) observed hypo-activation in children with dyslexia during addition and subtraction in the left supramarginal gyrus, a region that has been found to be associated with retrieving arithmetic facts in previous studies (see e.g., Chang et al., 2015; Cho et al., 2012; Davis et al., 2009; Peters et al., 2016).

There are currently no studies that have looked into the neural correlates of the comorbidity between dyslexia and dyscalculia.

In the current study, we directly compared for the first time the neural correlates of dyslexia, dyscalculia and comorbid dyslexia/dyscalculia. Children performed an arithmetic task inside the MRI scanner in which we manipulated presentation format (dot arrays, Arabic digits or number words). This manipulation was chosen because we wanted to maximize the chance of finding group differences between the different learning disorders under study. Specifically, we expected children with dyscalculia to perform more poorly on all conditions, as they all included numerical information and arithmetic. On the other hand, we expected the children with dyslexia to perform more poorly than controls only on symbolic formats, in particular the number words condition, in view of their poor reading skills. At the neural level, we predicted differences between dyscalculia and controls throughout the abovementioned described arithmetic network for all task conditions. For the children with dyslexia, we predicted differences in temporoparietal regions, such as angular and supramarginal gyri, and inferior frontal areas compared to controls in the symbolic but not non-symbolic task conditions, given the involvement of these regions in reading and in the verbal components of arithmetic (Dehaene et al., 2003; Martin et al., 2015).

Three types of analyses were used to gain more insight into the differences and similarities in the neurobiological correlates of dyslexia and dyscalculia. First, we used whole brain univariate analyses to check for hypo- or hyper-activation in the groups under study. Second, we used multivariate subject classification analyses to investigate whether children with dyslexia or dyscalculia showed similar neural activation patterns compared to typically developing children and compared to each other. Finally, we used multivariate subject generalization analyses to directly and statistically test the dissimilarity and/or similarity of the recruited neural activation patterns of children with dyslexia, dyscalculia and comorbid dyslexia/dyscalculia.

## 2 Materials and methods

### 2.1 Participants

Participants were 62 children (34 male) aged 9 to 12 years old (*M* = 10.83 years, *SD* = 0.83). All children with specific learning disorders included in the study (*n* = 39) received a formal diagnosis of a specific learning disorder by an experienced clinician in accordance with DSM-V (American Psychiatric Association, 2013) standards. These criteria involve the presence of persistent (i.e., longer than 6 months) deficits in arithmetic (dyscalculia) and/or reading ability (dyslexia) with scores of at least 1.5 standard deviations below the population mean for age, in the absence of intellectual disabilities, and in spite of scholastic opportunities and remediation. These children were further classified into three groups, depending on their specific diagnosis: children with dyslexia (DL, *n* = 19), children with dyscalculia (DC, *n* = 11), and children with comorbid dyslexia/dyscalculia (DLDC, *n* = 9). These groups of children with specific learning disorders were matched on age to a sample of typically developing children (TD, *n* = 23) without any history of learning difficulties. The data of all TD children were previously reported by Peters et al. (2016). Children were recruited from all over Flanders via schools, speech therapists, and online advertisement. None of the children had been diagnosed with additional developmental disorders (e.g., ADHD, autism), and none of them reported a history of psychiatric or neurological illness. All children had normal or corrected-to-normal vision, their parents gave written consent, and they were paid for their participation. The study was approved by the Medical Ethical Committee of KU Leuven.

**Fig. 1:**
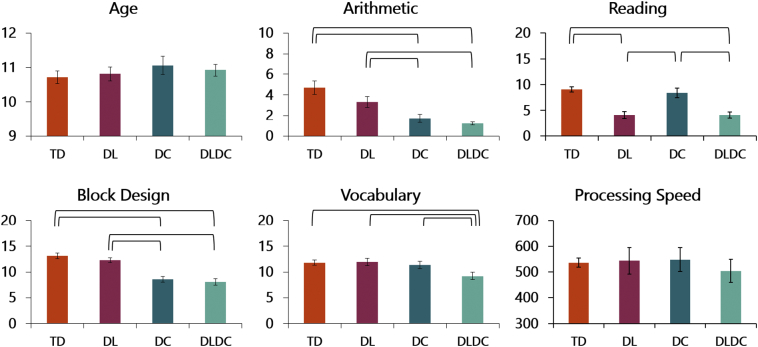
Standardized assessments per group. TD = typically developing, DL = isolated dyslexia, DC = isolated dyscalculia, DLDC = comorbid dyslexia/dyscalculia. Age is depicted in years, and arithmetic scores are deciles scores (*M* = 5). For reading, the mean of the standardized scores for the One Minute Test and the Klepel was used, scores for Block Design and Vocabulary are standardized scores as well (*M* = 10, *SD* = 3; Kort et al., 2005). Finally, processing speed is depicted in reaction time (in ms). Error bars represent the standard error of the mean, and means connected by brackets differed significantly on a *p* < 0.05 level.

We validated the clinical diagnoses by administering additional standardized tests for arithmetic and reading ability, as well as intelligence and processing speed (see Fig. 1 for the descriptive statistics for all four groups). Arithmetic ability was measured using the Tempo Test Arithmetic (TTA; de Vos, 1992), a standardized, five minute paper-and-pencil task that consists of addition, subtraction, multiplication and division problems. The assessment of reading ability consisted of the standardized One Minute Test (OMT; Brus and Voeten, 1979), in which children were asked to read aloud as many words correctly as possible within one minute, and the standardized Klepel (Van den Bos et al., 1994), a timed pseudo-word reading test in which was registered how many non-words a child could read aloud within two minutes. These standardized tests are included in the diagnostic protocol that is widely used in our country to diagnose dyscalculia and dyslexia. Intelligence was measured using the Vocabulary and Block Design subtests of the Dutch Wechsler Intelligence Scale for Children, Third Edition (WISC-III-NL; Kort et al., 2005). Finally, a control measure of processing speed was obtained using a reaction time task where children were asked to indicate, as fast as possible, which of two simultaneously presented figures was colored in white (Bellon et al., 2016).

ANOVAs with the presence of dyscalculia and the presence of dyslexia as between-subject factors were performed on the descriptive measures of all children that were finally included in the study (*N* = 52; see below for reasons for exclusion; see Fig. 1). These analyses showed that the four groups were matched on age. Children with dyscalculia (DC + DLDC) performed worse on the Tempo Test Arithmetic compared to children without dyscalculia (TD + DL), while children with isolated dyslexia (DL) did not differ from typically developing children (TD). Turning to reading ability, children with dyslexia (DL + DLDC) scored lower than children without dyslexia (TD + DC). All children scored within the normal range on the intelligence subtests, although children with dyscalculia (DC + DLDC) performed significantly more poorly than children without dyscalculia (TD + DL) on Block Design, a finding that has been observed in earlier studies (e.g., Berteletti et al., 2014; Kucian et al., 2011). On Vocabulary, children with comorbid dyslexia/dyscalculia scored lower than children from the three other groups, but their scores were close to the population average (Kort et al., 2005), indicating that their intellectual abilities were within the normal range. Finally, analyses showed that there were no group differences on our measure of processing speed.

### 2.2 Imaging study

#### 2.2.1 Imaging task

The arithmetic task reported previously by Peters et al. (2016) was performed by the children in the scanner. In this task, children were asked to subtract numbers below 10 and to indicate whether or not the solution was equal to a reference magnitude. This reference changed according to the run and was either 4 or 5 (presented in the fixed order of [4 5 4 5]), to allow for sufficient variation in the task. The format in which the numbers were presented varied, resulting in three format conditions: dot arrays, Arabic digits and number words. Fixation blocks and format blocks were alternated and lasted 15 s each. A format block comprised a presentation of the reference magnitude in the respective format (900 ms), and three trials consisting of a short fixation (300 ms) and a subtraction item (4400 ms). This paradigm resulted in the presentation of 12 trials per format in each run (four blocks per format, three subtraction items per block). Children performed four runs of this task.

**Fig. 2:**
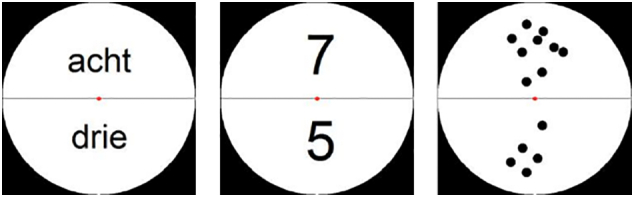
Examples of stimuli presented as number words, Arabic digits and dot arrays.

**Fig. 3:**
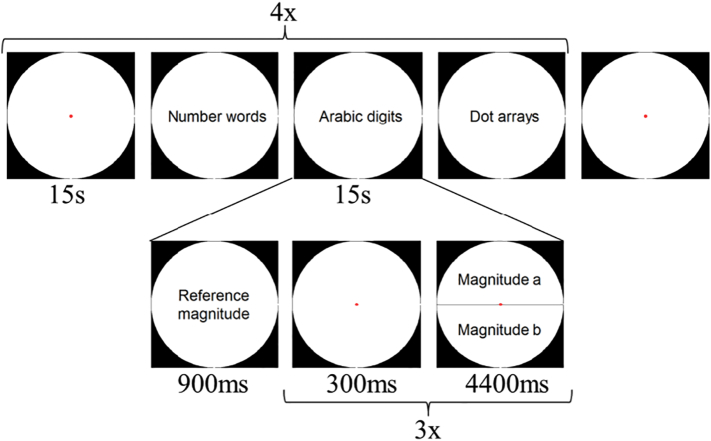
Schematic overview of the arithmetic task.

Subtraction items were presented in two halves of a white circle on a black background. Children were asked to subtract the number in the lower half of the circle from the number in the upper half (see Fig. 2), and to respond using two response buttons on a response box. All stimuli were created using an adapted version of a Matlab script (Dehaene et al., 2005) and were controlled for parameters such as total area and item size (for the dot arrays) and amount of visual information (i.e., number of black pixels) by varying the font size and the position of the digits and number words within the circle. The design of the task is illustrated in Fig. 3.

#### 2.2.2 Scanning parameters

Imaging data were collected via a 3T Philips Ingenia CX Scanner, at the Department of Radiology of the University Hospital in Leuven, with a 32-channel head coil and an EPI sequence (52 slices, 2.19 × 2.19 × 2.2 mm voxel size, interslice gap 0.3 mm, TR = 3000 ms, TE = 29.8 ms, flip angle = 90 degrees, 96 × 95 acquisition matrix). Furthermore, a high-resolution T1-weighted anatomical image (182 slices, resolution 0.98 × 0.98 × 1.2 mm, TE = 4.6 ms, 256 × 256 acquisition matrix) was acquired for each participant. Stimuli were displayed using PsychToolbox 3 (Brainard, 1997) and presented via a projector (NEC Display Solutions) onto a screen located approximately 46 cm from participants' eyes, which was visible via a mirror attached to the head coil.

### 2.3 Procedure

Data collection took place in two separate sessions. During the first session, the standardized behavioral assessment was carried out. Children were also intensively informed on the scanning procedure, and trained via a mock scanner in an environment that resembled the scanner environment as best as possible. The children practiced one run of the task in the mock scanner, while the noise of the scanner was simulated. During the second session, brain imaging data were collected at the University Hospital in Leuven. First, data were collected while children performed four runs of the arithmetic task. Second, the T1 anatomical image was acquired. Despite training with the mock scanner, three children (2 DL, 1 DLDC) were not comfortable enough in the scanning environment to successfully complete the scanning protocol. The behavioral data of these three children were not included in any of the analyses.

### 2.4 Analyses

#### 2.4.1 Behavioral analyses

Behavioral data were analyzed using SPSS (IBM SPSS Statistics 23; IBM Corp., Chicago, IL, USA). A Bonferroni correction was applied in all analyses to control for multiple comparisons. Trials in which participants did not respond, or responded too late due to the time limit (i.e., 4400 ms) were excluded from the accuracy scores and reaction times.

### 2.5 fMRI preprocessing and analyses

For the analyses of the imaging data, the Statistical Parametric Mapping software package (SPM8, Wellcome Department of Cognitive Neurology, London) was used. To avoid a decrease in data quality due to excessive motion during scanning, all runs in which participants showed excessive movement were removed from all analyses, which is exactly the same procedure as used earlier by Peters et al. (2016). Specifically, two different motion criteria were used. All runs in which a movement of more than one voxel size (=2.2 mm) in either direction on two consecutive scans was found, were discarded from the analyses. Furthermore, runs in which a Euclidean distance measure, which is an additive measure of the amount of motion in all directions from one time point to another, exceeded one voxel size were also removed. Participants with less than half of the runs remaining were excluded from all analyses (behavioral and fMRI). This criterion led to the discarding of seven additional participants (1 TD, 3 DL and 3 DC), leading to a final sample of 52 children (22 TD, 14 DL, 8 DC and 8 DLDC). Of these remaining participants, 10.33% of the runs were excluded from the analyses due to motion. Importantly, the four groups of children did not differ in degree of motion after these measures to remove excessive motion (*F*(3,48) = 1.39, *p* = 0.26), nor on cumulative motion over the entire run (*F*(3,48) = 1.89, *p* = 0.14). Finally, the groups did not differ in the average number of runs that were included per participant (*F*(3,48) = 2.07, *p* = 0.12).

Functional images were corrected for slice-timing differences and for head motion artifacts by realigning all images to the first image. Functional images were co-registered to the anatomical image. Both functional and anatomical images were normalized to the standard Montreal Neurological 152-brain average template, and finally, functional images were smoothed using a Gaussian kernel of 10 mm full-width at half maximum (FWHM). The decision for the use of this smoothing kernel was based on Mikl et al. (2008), who found that the optimal kernel for group inference is 8 mm FWHM, but that higher kernels are better when there are fewer subjects in the study. The effect of the experimental conditions per voxel was estimated using boxcar functions corresponding to the block length. Motion realignment parameters were included as regressors of no interest in the general linear models, to further control for variation due to movement artifacts. Contrasts between each format and fixation resulting in voxel-wise *t*-statistics maps were calculated per participant.

To statistically test which brain regions were activated more for one group of children compared to another, a whole brain ANOVA with dyslexia and dyscalculia as between subject factors was performed on the imaging data, for each condition versus fixation. A false discovery rate (FDR, *p* < 0.05) correction was applied at the whole brain level to correct for multiple comparisons.

Multivariate subject classification analyses were used to investigate whether we could classify children into their diagnostic group based on their neural activation patterns for each format versus fixation. In order to guarantee that between-subject variability in BOLD response would not account for the results obtained using this multivariate analysis, activation levels were mean-centered for each individual subject. Unlike in the full factorial ANOVA, this analysis does not use a voxel-to-voxel activity based comparison, but rather compares spatial patterns of activation in selected regions of interest (ROIs). As arithmetic recruits a large, whole brain network (see above), five large ROIs were selected with anatomical masks from the WFU PickAtlas: whole brain grey matter, occipital lobe, parietal lobe, frontal lobe and temporal lobe. Using the same approach, seven smaller ROIs were selected based on the arithmetic network described above (Menon, 2015; Peters and De Smedt, 2017) and anatomically delineated: superior and inferior parietal lobules, inferior and superior frontal gyri, angular gyrus, supramarginal gyrus and fusiform gyrus. A leave-pair-out-cross-validation (LPOCV; Ung et al., 2014) was run on the beta weights of the contrast of each condition (dots, digits and number words) versus the fixation condition. A linear classifier was trained on distinguishing between the participants of two groups, except one randomly selected pair of subjects (one from each group). The classifier was subsequently tested on the remaining pair of subjects. This procedure was repeated until each participant was left out of training once. This LPOCV-procedure was run 1000 times. Classification accuracies were then averaged over these repetitions. As our group sizes differed between groups, the smallest group size was used. Participants from the larger group were randomly left out of the LPOCV-iteration to match the group size of the smaller group. To determine the critical classification value, a Monte Carlo permutation test was performed (Mourão-Miranda et al., 2005). In this test, category labels of the training set were randomly permuted, followed by 1000 iterations of the LPOCV-procedure. Subsequently, the significance border was set using the 95% confidence interval cutoff on these 1000 iterations. This analysis was performed six times per ROI: to differentiate TD from DL + DLDC, TD from DC + DLDC, TD from DL, TD from DC, TD from DLDC and finally DL from DC.

We also applied a multivariate subject generalization analysis to directly and statistically assess the similarity of the neural activation patterns of groups of children with learning disorders. The LPOCV-procedure from the subject classification analysis was used, with the exception that in this analysis, the model was trained on differentiating TD children from one learning disorder group (e.g., DL), and tested on differentiating TD children from another learning disorder group (e.g., DC). Generalizing over two groups always occurred bi-directionally: The model was trained on DL and tested on DC, but in addition also trained on DC and tested on DL. The average generalization accuracy of both directions is presented. This generalization will only be significant if neural activation patterns of the DL and DC groups are very similar, fooling the model into believing that the activation patterns belong to the same group. Again, a Monte Carlo permutation test was performed to determine the significance cutoff criterion. This analysis was performed three times per ROI: once to generalize between DL and DC, once to generalize between DL and DLDC, and once to generalize between DC and DLDC.

## 3 Results

### 3.1 Behavioral results

**Fig. 4:**
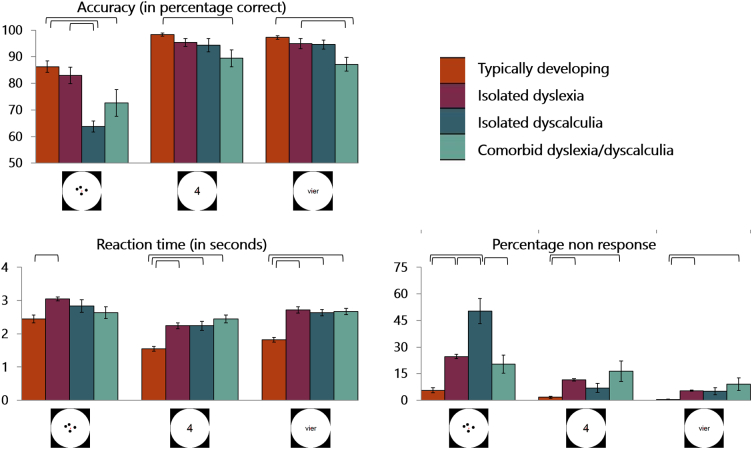
Mean accuracy, reaction time (in seconds) and percentage non-response on the arithmetic task per format (dots, digits and words) and per group. Error bars represent the standard error of the mean. Means connected by brackets differed significantly on a *p* < 0.05 level.

To look into the behavioral results of the arithmetic task, mixed ANOVAs with the presence of dyscalculia and the presence of dyslexia as between-subject factors, and format (dots vs. digits vs. number words) as within-subject factor were performed on accuracies, reaction times and percentages of non-response (see Fig. 4). Details on main and interaction effects per analysis are presented in Table 1.

**Table 1:** Main effects and interaction effects of the arithmetic task.

		Accuracy		Reaction time		Non response	
	*df*	*F*	*p*	*F*	*p*	*F*	*p*
Format	2,96	106.23	<0.001	43.32	<0.001	51.31	<0.001
DL	1,48	1.39	0.244	19.38	<0.001	1.74	0.194
DC	1,48	27.65	<0.001	9.50	0.003	18.98	<0.001
Format × DL	2,96	4.48	0.014	2.45	0.091	6.80	0.002
Format × DC	2,96	11.06	<0.001	6.92	0.002	9.15	<0.001
DL × DC	1,48	0.25	0.619	16.61	<0.001	13.88	<0.001
Format × DL × DC	2,96	5.51	0.005	1.14	0.323	22.04	<0.001

Regarding the accuracy scores, a main effect of format was found. Children performed worse on dot arrays than on Arabic digits and number words (both *p*s < 0.001), whereas the performance on digits and words did not differ (*p* = 0.47). Also, children with dyscalculia (DC + DLDC) performed worse than children without dyscalculia (TD + DL). On the other hand, children with (DL + DLDC) and without dyslexia (TD + DC) did not differ in their performance on the task. There was a significant interaction between format and dyscalculia, which can be explained by the larger difference between children with (DC + DLDC) and without dyscalculia (TD + DL) in performance on dots than on digits and words. The interaction effect between format and dyslexia on the other hand can be explained by the fact that, due to the low performance of children with isolated dyscalculia (DC) on the dot format, children with dyslexia (DL + DLDC) actually performed *better* on dot arrays compared to children without dyslexia (TD + DC), while children with dyslexia (DL + DLDC) performed *worse* on digits and number words compared to children without dyslexia (TD + DC). Finally, the interaction effect between dyslexia and dyscalculia was not significant, indicating that reading ability did not influence the finding that children with dyscalculia (DC + DLDC) performed worse than children without dyscalculia (TD + DL).

Turning to the reaction times, we found a main effect of format: Children responded faster to digits than to words (*p* < 0.001), and faster to words than to dots (*p = 0*.002). Furthermore, children without dyscalculia (TD + DL) responded faster than children with dyscalculia (DC + DLDC). Similarly, children without dyslexia (TD + DC) responded faster than children with dyslexia (DL + DLDC). Finally, the significant interaction effect between dyslexia and dyscalculia (*F*(1,48) = 16.61, *p* < 0.001) showed that typically developing children were faster in responding compared to all children with learning disorders. This interaction effect also showed under-additivity for the comorbid group (DLDC), indicating that they were less impaired compared to the sum of the isolated groups.

Since the task performed in the scanner was timed, we also looked into the percentage of items that subjects were not able to solve within the given time limit. A significant main effect of format was present. Subjects responded to fewer dot items than digit items (*p* < 0.001), and to fewer digit items than number words items (*p* = 0.004). Furthermore, children with dyscalculia (DC + DLDC) responded to fewer items than children without dyscalculia (TD + DL), whereas children with dyslexia (DL + DLDC) responded to an equal number of items compared to children without dyslexia (TD + DC). Significant interaction effects between format and dyscalculia and format and dyslexia however, showed that the difference in non-response on dots was larger in children with dyscalculia (DC + DLDC) compared to those without dyscalculia (TD + DL). Children with dyslexia (DL + DLDC), on the other hand, showed higher non-response on digits and number words compared to children without dyslexia (TD + DC), but these groups did not differ in terms of their non-response to the dots. Similarly to the accuracy scores, this interaction effect appeared to be driven mostly by the very high percentage of non-response for dots for children with isolated dyscalculia (DC). Finally, the interaction effect between dyslexia and dyscalculia reflected the fact that, across all formats, children with isolated dyscalculia (DC) solved the fewest items, and typically developing children the most.

In summary, the impairments in children with dyscalculia (DC + DLDC) were most pronounced in the dot condition, where they were less accurate and more often late in responding compared to children without dyscalculia (TD + DL). All children with learning disorders were slower in responding compared to typically developing children.

### 3.2 Imaging results

#### 3.2.1 Univariate analyses

**Fig. 5:**
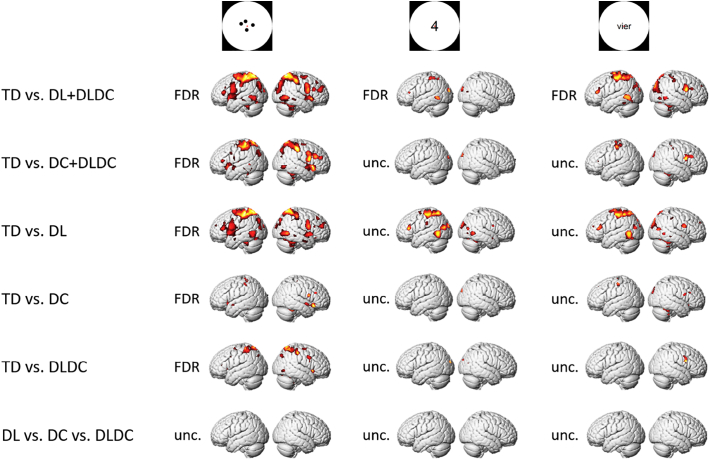
Activation patterns of all three formats (dot arrays, Arabic digits and number words) of the arithmetic task versus fixation, of TD vs. DL + DLDC, TD vs. DC + DLDC, TD vs. DL, TD vs. DC, TD vs. DLDC and DL vs. DC vs. DLDC. Activation patterns are shown uncorrected (*p* < 0.001) only if no activation clusters survived FDR-correction (*p* < 0.05).

Whole brain ANOVAs with dyslexia and dyscalculia as between-subject factors were performed on all formats versus fixation (see Fig. 5). These analyses showed that typically developing children (TD) elicited more activation for dot arrays compared to children with dyscalculia (DC + DLDC) and children with dyslexia (DL + DLDC), and these effects were spread out over a whole brain network, in frontal, parietal, temporal and occipital regions. For the Arabic digits, we also found higher activation levels for typically developing children compared to children with dyslexia (DL + DLDC) in a smaller set of regions, which included the left posterior and inferior parietal areas, bilateral cuneus, left middle temporal gyrus and left inferior frontal gyrus. The comparison between typically developing children and children with dyscalculia (DC + DLDC) showed a similar pattern of results at the uncorrected level (*p* < 0.001), but this pattern did not survive FDR-correction. Similar results were found for the number words: Typically developing children showed higher activation levels compared to children with dyslexia (DL + DLDC) in left posterior and inferior parietal areas, bilateral cuneus and inferior and middle occipital areas, bilateral middle temporal gyrus and bilateral inferior frontal gyrus. This pattern of findings was also present for typically developing children versus children with dyscalculia (DC + DLDC), albeit only at an uncorrected level (*p* < 0.001). For all three formats, there were no brain regions that were activated more by children with a learning disorder compared to typically developing children, also not at an uncorrected level (*p* < 0.001). We would like to emphasize that these results were not driven by the inclusion of children from the comorbid group (DLDC) in all contrasts. As can be seen in Fig. 5, the group of children with isolated dyslexia (DL), which contains the largest number of children of any group of children with learning disorders (DL, DC and DLDC) showed the strongest effect.

#### 3.2.2 Subject classification analyses

**Fig. 6:**
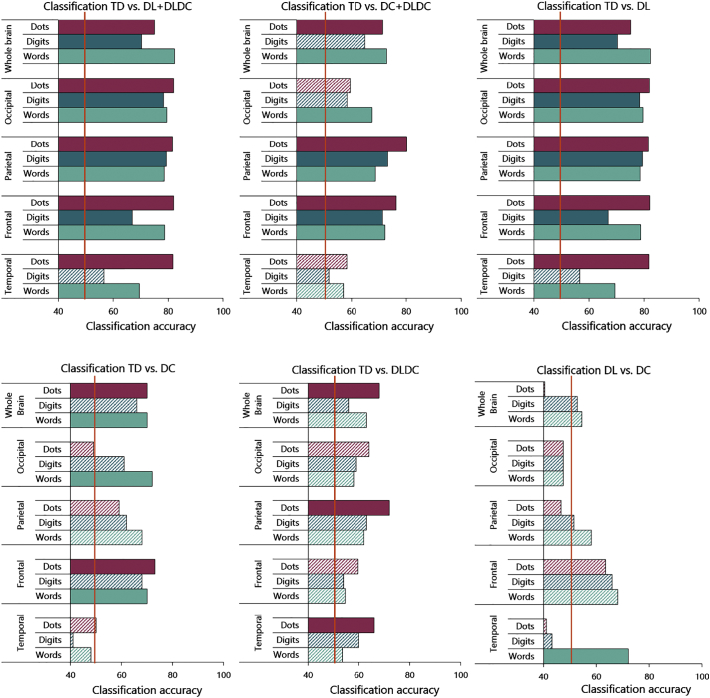
Classification accuracies per format (dots, digits and words) and per large ROI (whole brain, occipital, parietal, frontal and temporal lobes) for the arithmetic task. Accuracies that reached significance are solidly filled, and chance level (50%) is indicated with an orange line.

As an additional statistical test of differences between subject groups, we performed multi-voxel subject classification analyses. These analyses (see Fig. 6) allowed us to investigate whether we could classify children into their respective group, based on their neural activation *patterns* during arithmetic. This was done for each format vs. fixation contrast separately.

The classification analysis differentiating typically developing children from children with dyslexia (DL + DLDC), showed that for dots, digits and words, and in each large ROI (whole brain, occipital lobe, parietal lobe, frontal lobe and temporal lobe) we were able to significantly differentiate typically developing children from children with dyslexia (DL + DLDC) based on their neural activation patterns. The only region in which the classification during Arabic digits did *not* reach significance, was the temporal lobe. In other words, in the temporal lobe, the neural activation patterns elicited by Arabic digits were insufficiently distinct to categorize typically developing children from children with dyslexia (DL + DLDC). In all other large ROIs however, the neural activation patterns elicited by all formats of our task allowed a trained model to accurately categorize children into typically developing children vs. children with dyslexia (DL + DLDC). We also ran this analysis in a series of smaller ROIs (see Appendix A for the full results), and those results were similar.

For the classification analysis differentiating between typically developing children and children with dyscalculia (DC + DLDC), a similar pattern of findings was found in parietal and frontal lobes: Classification was significantly accurate for dots, digits and words. At the whole brain level, classification was significant for dots and words, in the occipital lobe for words only, but in the temporal lobe, classification accuracies did not reach significance for any of the formats. Our trained classifier was thus able to correctly categorize typically developing children and children with dyscalculia (DC + DLDC) based on the neural activation patterns elicited by all formats in frontal and parietal areas. We again ran this analysis in the smaller ROIs, and found similar, yet less strong results (see Appendix A).

These findings suggest that children with learning disorders showed somewhat distinct neural activation patterns compared to typically developing children. It is again important to note that also in these series of analyses, results were not driven by the inclusion of children from the comorbid group (DLDC) in the analyses. As can be seen in Fig. 6, distinguishing between typically developing children and children with isolated dyslexia (DL) showed higher classification accuracies than distinguishing typically developing children from children with isolated dyscalculia (DC) or comorbid dyslexia/dyscalculia (DLDC).

Finally, the classification analysis differentiating between children with dyslexia and children with dyscalculia (DL vs. DC) did not reach significance in any of the regions for any of the formats, except for number words in the temporal cortex. This suggests that the neural activation patterns of children with learning disorders during arithmetic were very similar in the large ROIs and therefore difficult to distinguish from one another by a trained classifier.

Subject classification analyses were also performed within the smaller ROIs mentioned above. Although the classification accuracies in those areas were lower, they followed the same patterns of results compared to the analyses presented here (see Appendix A and Fig. A.1).

#### 3.2.3 Subject generalization analyses

An exploratory, visual inspection of the whole-brain univariate analyses in Section 3.2.1 (Fig. 5) suggested that the regions activated more by typically developing children compared to the different groups of children with learning disorders were surprisingly similar in anatomical terms. To test these main effects, we had a large group of subjects because the analysis pooled subjects across the specific groups. For example, to test for the effect of dyslexia, the analysis pooled across the group with isolated dyslexia (DL; *n* = 14) and the group with co-morbidity (DLDC; *n* = 8). It remains to be determined whether there are interactions between these effects, for example, whether the effect of dyslexia depends upon the presence of dyscalculia. To test this, we performed direct comparisons of children with specific combinations of learning disorders. These comparisons revealed no brain regions that were activated more by children from one group compared to another, also not on an uncorrected level (*p* < 0.001; see Fig. 5, DL vs. DC vs. DLDC).

However, as there was only a small number of subjects in some of the groups with learning disorders, the direct univariate comparisons of the activation patterns of the different groups were exploratory and underpowered, in particular because similarity in activation differences would amount to a null result of *no* differences between learning disorders. The same holds for the classification analysis differentiating between children with dyslexia and children with dyscalculia, where neural similarity would be reflected as a null result. Furthermore, and more crucially, even *if* between-group differences had been found with this relatively small subject sample, these direct contrasts would not show the magnitude of these differences relative to the similarities between learning disorders. It could be that potentially observed differences, as may have been revealed in other studies, are very small relative to the existing similarities. To answer these questions and to directly statistically test the degree of similarity suggested by the univariate and subject classification analyses, we performed multi-voxel subject generalization analyses, in which we directly tested the ability of the trained multi-voxel classifiers to generalize from one learning disorder to the other.

**Fig. 7:**
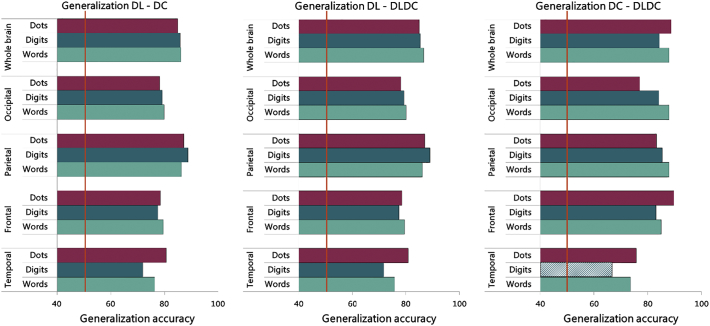
Generalization accuracies per format (dots, digits and words) and per large ROI (whole brain, occipital, parietal, frontal and temporal lobes) for the arithmetic task. Accuracies that reached significance are solidly filled, and chance level (50%) is indicated with an orange line.

The outcome of these multi-voxel subject generalization analyses are depicted in Fig. 7. These analyses showed that a classifier trained to distinguish between typically developing children and a group of children with one learning disorder (e.g., DL) and tested on differentiating typically developing children from a group of children with a different learning disorder (e.g., DC) and vice versa was significantly accurate for all formats and in all large ROIs (see Fig. 7). Thus, overall, the atypical activation patterns observed in isolated dyslexia (DL) generalize significantly to the atypical activation patterns observed in isolated dyscalculia (DC), and vice versa. Maybe less surprisingly, the generalization also works from groups with a single isolated learning disorder to the comorbid group (DLDC) and vice versa, with exception of digits in temporal cortex for generalization between DC and DLDC.

These results clearly show that the neural activation patterns during this task of children with learning disorders (DL, DC or DLDC) were sufficiently similar to be mistaken by the classifier for activation patterns from children of a different learning disorder group. This was true independent of the format in which the arithmetic stimuli were presented. This indicates that the neural activation patterns were similar across learning disorders, yet they were clearly distinct from the neural activation patterns of typically developing children.

It is important to emphasize that these generalization analyses were performed in two directions: training on specific disorder X and testing on disorder Y, and vice versa. The average generalization accuracy of both directions is presented here. For that reason, the number of subjects with a disorder on which the generalization is tested was equal to the sum of subjects with the two disorders (and of course in addition also to an equal number of control subjects). Thus, even though the final fMRI dataset only included data from 14 children with isolated dyslexia and 8 children with isolated dyscalculia, the generalization of the difference between each of these disorders and controls was tested on 22 children with a disorder and an equally large group without a disorder. From that point of view, the conclusion of highly significant generalization and thus surprisingly high commonalities between the disorders was based upon a very reasonable sample size.

These multivariate subject generalization analyses were also performed within the smaller ROIs. Generalization accuracies in all ROIs and over all formats pointed towards very similar neural activation patterns for children with learning disorders in smaller ROIs as well. A full overview of the generalization analyses performed in these smaller ROIs can be found in Appendix A (see Fig. A.2).

In order to validate that these findings were not solely driven by the task used, all participating children also performed a reading task in the scanner (see Appendix B). Similar analyses were performed on the data of that task, and those analyses led to similar, yet less powerful conclusions. This might be because even more children had to be excluded from the analyses due to excessive motion (i.e., 2 TD, 6 DL, 4 DC and 3 DLDC, leading to a final sample of 45 children), which was due to the fact that the task occurred at the end of our imaging paradigm (see Appendix B). In this reading task, children with learning disorders showed hypo-activation and distinct neural activation patterns compared to typically developing children, and the neural activation patterns of children with different learning disorders were remarkably similar. A full overview of the task design (see Fig. B.1) and results can be find in Appendix B (see Table B.1 and Figs. B.2 to B.5).

## 4 Discussion

The current study investigated the neural correlates of arithmetic in children with dyslexia, dyscalculia, comorbid dyslexia/dyscalculia and typically developing children. This was the first study in which the neural arithmetic network was directly compared between different groups of children with learning disorders and controls. At the behavioral level, we found that children with dyscalculia (DC + DLDC) performed more poorly on dot arrays compared to children without dyscalculia (TD + DL), and that all children with learning disorders were slower in responding compared to typically developing children. At the neural level, our findings point to a surprising degree of neural similarity between the different learning disorders during an arithmetic task.

We observed hypo-activation for all children with learning disorders compared to typically developing children throughout the brain, which is in line with some earlier studies in dyscalculia (see e.g., Ashkenazi et al., 2012; Berteletti et al., 2014), yet not with others, such as Rosenberg-Lee et al. (2015), in which hyper-activation was reported for children with dyscalculia compared to healthy controls (see also Davis et al., 2009). However, none of the previous imaging studies used clinically validated diagnoses to categorize children into groups, and paradigms used in the various studies differed vastly. These differences in participants and methodology could possibly account for the discrepancies in results between the current study and previous studies.

Arithmetic difficulties, particularly with fact retrieval, are also very common in children with dyslexia (Göbel, 2015; Simmons and Singleton, 2008; Träff and Passolunghi, 2015). Also in the current study, children with dyslexia were slower in responding during the arithmetic task (see Fig. 4 and Table 1), despite arithmetic abilities similar to those of typically developing children (see Table 1). Thus far the only neuroimaging study investigating arithmetic in children with dyslexia (Evans et al., 2014), reported hypo-activation in left supramarginal gyrus in children with dyslexia compared to typically developing children during addition and subtraction. These results are in line with the hypo-activation found for children with dyslexia during arithmetic in the current study, yet the current data indicate that this lowered activity is more widespread than the supramarginal gyrus.

The current study is the first neuroimaging study that included and directly compared children with dyslexia, children with dyscalculia and children with comorbid dyslexia/dyscalculia. The whole brain, univariate analyses did not reveal any regions recruited more by one group of children with learning disorders than by another group. The absence of group differences between children with dyslexia and children with dyscalculia could however potentially reflect a power issue due to the rather small sample sizes. Therefore, we also performed multi-voxel subject classification and generalization analyses, to directly, statistically test for neural similarity in recruited neural activation patterns over groups.

The subject classification analyses showed that the neural activation patterns of typically developing children were sufficiently distinct from the neural activation patterns of children with dyslexia and, to a lesser extent, of children with dyscalculia, for a trained model to classify children with dyslexia, children with dyscalculia and typically developing children correctly. Furthermore, the neural activation patterns of children with dyslexia and children with dyscalculia were difficult to distinguish by a trained classifier. The subject generalization analyses further confirmed these findings and, critically, showed that the neural activation patterns of children with different learning disorders (dyslexia, dyscalculia and comorbid dyslexia/dyscalculia) were sufficiently similar to allow a trained classifier to generalize from one learning disorder to the other. It is important to stress that we did not only find this neural overlap in larger regions of interest (e.g., frontal and parietal lobe), but also in smaller brain regions that are directly implicated in arithmetic (Menon, 2015; Peters and De Smedt, 2017). Both on a large, as well as on a smaller scale, this neural similarity thus appeared to hold. It is also remarkable that the generalization classification accuracies were not lower than the within-group subject classification accuracies. This further suggests that the individuals from the different learning disorder groups were very similar in how they differed from typically developing children in terms of their brain activity. It is also important to note that these results are *by no means* null-results potentially caused by power issues, but significant, statistical tests of similarity between groups of children with different learning disorders. Furthermore, due to the bi-directional nature if this analysis, group sizes in the subject generalization were very reasonable. We are not excluding the possibility of finding group differences with a larger sample of children, but would like to emphasize that nonetheless, the neural similarity between children with learning disorders, which was statistically demonstrated here, is surprisingly substantial, and more pronounced and convincing than potential group differences would be.

We would like to stress that the subject generalization analyses were done specifically on the groups with an isolated disorder (i.e., dyslexia and dyscalculia), excluding the comorbid group. It is thus unlikely that the significant and robust classification accuracies were driven by the inclusion of children with comorbid dyslexia/dyscalculia in both analyses. In addition, but not unexpectedly, generalization was also possible from the isolated dyslexia or dyscalculia group to the comorbid dyslexia/dyscalculia group. These results showed that, at the neural level, children with comorbid dyslexia/dyscalculia vastly resembled both children with dyslexia-only and children with dyscalculia-only.

These remarkable results reflecting neural similarity between children with different learning disorders in the context of arithmetic, are strengthened by similar findings in the reading task (see Appendix B). Also in this task, children with learning disorders showed hypo-activation compared to typically developing children, which is in line with previous research on dyslexia (see Gabrieli, 2009 for a review). Furthermore, multivariate subject classification and generalization analyses showed similar results compared to in the arithmetic task, albeit less strong. This might be due to less power in the reading task due to the loss of additional data because of motion (see Appendix B).

It is important to note that both dyslexia and dyscalculia are very heterogeneous disorders. Literature has shown, for example, that not all children with dyslexia present with phonological deficits (Snowling, 2008), and that other neurocognitive correlates of dyslexia have been identified (e.g., temporal processing, working memory, visuospatial attention; e.g., Facoetti et al., 2000; Goswami, 2011; Smith-Spark and Fisk, 2007). Similarly, different clusters of behavioral characteristics have been reported in children with dyscalculia, which were not all characterized by deficits in number processing (e.g., Bartelet et al., 2014). It is also important to note in this context that proposed multiple deficit models (e.g., Pennington, 2006) assume that there are no core, isolated correlates for disorders, but rather that multiple interacting factors contribute to the existence of these disorders. Given the influence of multiple factors, it is unsurprising that highly variable phenotypes have been described for each learning disorder. Whether the neural similarities between groups of children with dyslexia and/or dyscalculia reported here would hold for all phenotypical expressions of these disorders, remains unclear. Unfortunately, our small group sizes and limited cognitive testing battery do not allow us to further look into the effect of within-disorder heterogeneity on children's neural correlates of arithmetic.

Research exploring the cognitive correlates of the comorbidity between dyslexia and dyscalculia has typically reported additive effects of dyslexia and dyscalculia in the comorbid groups (see e.g., Landerl et al., 2009). However, to date, there are no studies that have looked into the neural correlates of this comorbidity. Given the high degree of neural similarity between the dyslexia-only and dyscalculia-only groups, it is not surprising that it was impossible to distinguish the comorbid group from both other groups of children with learning disorders at the neural level. It is currently unclear how this neural similarity, in the context of both the arithmetic and the reading task, is associated with observed additive effects of comorbidity at the behavioral level. In all, there is a clear need for more research on the specific nature and correlates of the comorbidity between these two learning disorders.

What might account for these unexpected neural similarities across the neurodevelopmental learning disorders under study? First, the observed findings could reflect a task difficulty effect. As the analyses on the reaction time data revealed, all children with learning disorders were slower in responding compared to typically developing children, which could reflect an overall higher task difficulty level experienced by all children with learning disorders. However, in our behavioral measure of processing speed, we did not detect any group differences. Furthermore, in the most demanding format condition of the arithmetic task (dot arrays), the difference in activation levels between typically developing children and children with dyslexia and children with dyscalculia was more prominent in comparison to in the less demanding format conditions (digits and number words). These results could suggest that as task difficulty increases, children with learning disorders are less efficient in modulating neural activation in recruited neural networks. Future studies would benefit from using event-related designs, which would allow to discard incorrect trials, and trials on which the participant did not respond (in time).

Second, these results could reflect differences in the recruitment of domain-general resources, such as working memory. Research has shown that working memory is affected in both dyscalculia (Swanson and Beebe-Frankenberger, 2004) and dyslexia (Smith-Spark and Fisk, 2007). As we found similar results in both the arithmetic and the reading task, it is possible that task-independent correlates, such as working memory, rather than task-specific correlates influenced our findings.

Third, it could also be the case that the tasks used here (arithmetic and reading) lacked the specificity to pick up small subtle differences between children with learning disorders. Based on the current findings, we cannot exclude the possibility of specific neurobiological differences between dyscalculia and dyslexia, in addition to the shared atypical activation profile. It is possible that, for example, tasks tapping more directly into cognitive processes such as number processing or phonological processing, that have been repeatedly associated with dyscalculia and dyslexia, respectively (Butterworth et al., 2011; Wagner and Torgesen, 1987), could show neural differences between children with various learning disorders. However, the results presented here clearly show larger neural similarity between dyslexia and dyscalculia than previously assumed.

Finally, this pattern of findings could also be explained academic nature of both tasks. Previous research has shown that reading and arithmetic skills are correlated, likely due to the importance of reading skills in acquiring arithmetic knowledge (Fuchs et al., 2005, Fuchs et al., 2006; Hecht et al., 2001; Jordan et al., 2003). A study in monozygotic and dizygotic twins, has provided evidence in favor of the so called *generalist genes* hypothesis, which states that most genes associated with one academic skill (e.g., arithmetic) are also associated with another academic skill (e.g., reading), be it that some genes will have more specific effects (Haworth et al., 2009). Furthermore, a study by Docherty et al. (2010) found SNPs associated with both arithmetic and reading ability. These similar genetic influences are thus presumed to lie at the base of the development of (problems with) both reading and arithmetic (Krapohl et al., 2014; Light and DeFries, 1995; Mascheretti et al., 2014; Plomin and Kovas, 2005). This common genetic influence might affect the neurobiological origin of dyslexia, dyscalculia and comorbid dyslexia/dyscalculia in a similar way, which could result in comparable aberrant neural modulation during academic tasks in general, such as arithmetic and reading.

Additionally, we would also like to point out that the degree of similarity between dyslexia, dyscalculia and comorbid dyslexia/dyscalculia is also somewhat reflected in the DSM-V, as it speaks of subtypes of specific learning disorders with the same cognitive characteristics: difficulties in learning and using academic skills (American Psychiatric Association, 2013). The DSM-V's approach is more clinically oriented, and is likely based on the high comorbidity of problems in arithmetic and reading in clinical settings.

No matter how these factors work (together) to result in highly similar atypical patterns of neural activation in the two learning disorders, it remains that this high degree of neural similarity was unexpected given the literature on neurodevelopmental disorders, which is dominated by studies focusing upon single, isolated disorders. Note that our two experiments are very representative for the experiments that researchers would design to study either dyscalculia (arithmetic task) or dyslexia (reading task). In a typical isolated study on an isolated disorder, researchers would be tempted to consider their findings as specific to the targeted disorder. Our study shows that this tunnel vision is unwarranted. This is even more so because many studies in the literature have ignored comorbidity, and thus have included a less specific clinical group compared to our study.

Although we believe that our findings are extremely important as a benchmark to reconsider the dominant approach in the literature, much more work remains to be done. We do not exclude the possibility that, in addition to a shared atypical activation profile, there are also specific differences between dyslexia and dyscalculia that could be robustly found with very specific paradigms. However, this study shows that the degree of neural similarity between learning disorders is more pronounced than potential group differences might be. It is important to note that neural markers other than task-related brain activity might provide a different results. For example, it would also be interesting to look into (dis)similarities in neural connectivity between children with dyslexia, children with dyscalculia and children with comorbid dyslexia/dyscalculia. Previous research has reported hyper-connectivity between frontal and parietal areas in children with dyscalculia (Jolles et al., 2016; Rosenberg-Lee et al., 2015) and hypo-connectivity in children with dyslexia (see Vandermosten et al., 2012 for a review), but a direct comparison of connectivity between children with learning disorders has never been made. This represents an important area for future study. Finally, the comorbidity rates between other neurodevelopmental disorders, such as ADHD and autism, are also rather high (American Psychiatric Association, 2013). It would therefore be interesting, and as this study clearly shows, vital, to not only study the neurobiological origin of these developmental disorders in isolation, but to also be aware of potential neural similarities between different developmental disorders.

## Appendix A Supplementary data

Appendix A Multivariate analyses in smaller ROIs. Appendix A

Appendix B Reading task. Appendix B
