# Supplementary material for: Dyscalculia and dyslexia: Different behavioral, yet similar brain activity profiles during arithmetic
Source: Neuroimage Clin. 2018 Mar 4;18:663–74. doi: 10.1016/j.nicl.2018.03.003 (PMC5987869; doi:10.1016/j.nicl.2018.03.003)
Supplement: Appendix A — Multivariate analyses in smaller ROIs. [file mmc1.docx]

**Appendix A: Multivariate analyses in smaller ROIs**

In addition to performing multivariate subject classification and generalization analyses in large regions of interest comprising whole brain, occipital lobe, parietal lobe, frontal lobe and parietal lobe, we also performed identical analyses within seven smaller regions of interest, which were selected based on the literature (Menon, 2015; Peters & De Smedt, in press). These smaller ROIs included inferior parietal lobule (IPL), superior parietal lobule (SPL), supramarginal gyrus (SMG), inferior frontal gyrus (IFG), superior frontal gyrus (SFG), angular gyrus (AG) and fusiform gyrus (FG). These regions were delineated using anatomical masks available from the WFU PickAtlas. For the classification analyses, results are shown in Figure A.1; the results of the generalization analyses are presented in Figure A.2.


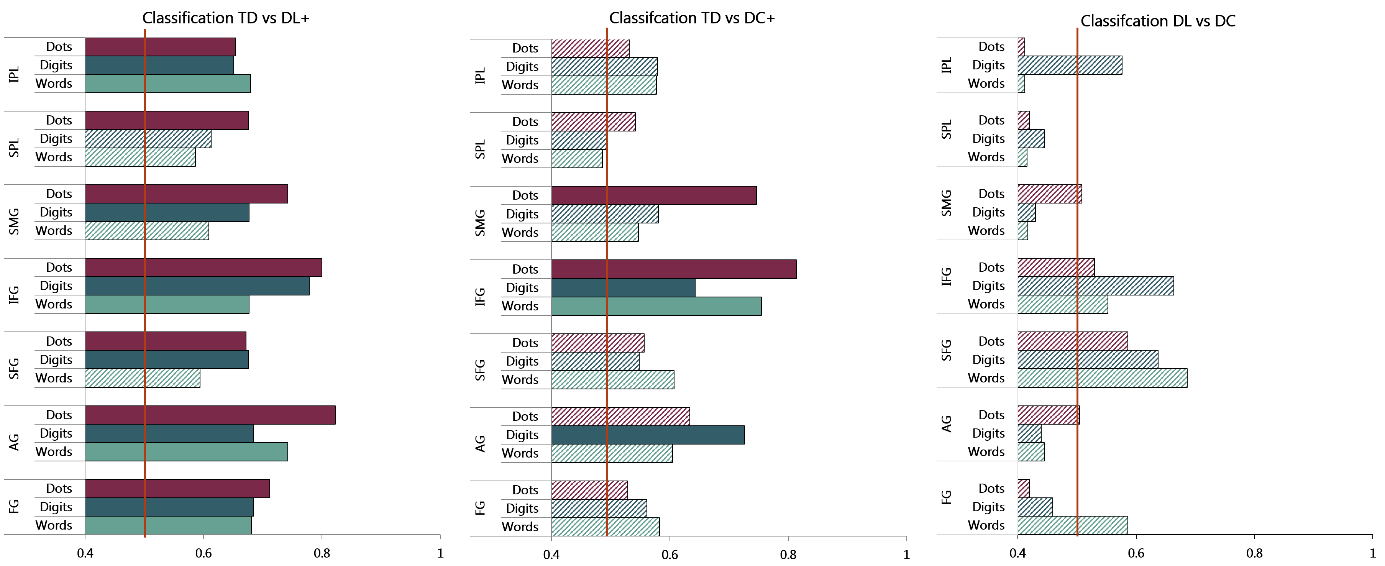


*Figure A.1*. Classification accuracies per format (dots, digits and words) and per smaller ROI. Accuracies that reached significance are solidly filled, and chance level (0.50) is indicated.

The classification analysis differentiating typically developing children from children with dyslexia showed that classification accuracies were significant for all formats in the inferior parietal lobule and in the inferior frontal, angular and fusiform gyri. Furthermore, classification for dots and digits was significant in the supramarginal and superior frontal gyri. Finally, classification for dots reached significance in the superior parietal lobule.

For the classification analysis differentiating between typically developing children and children with dyscalculia, the results were less convincing. Only in the inferior frontal gyrus, classification was significant for all formats. Furthermore, the model was able to distinguish between typically developing children and children with dyscalculia for dots in the supramarginal gyrus and for digits in the angular gyrus. None of the other classification accuracies reached significance. It is rather surprising that the model was not able to distinguish between typically developing children and children with dyscalculia in the delineated parietal areas, given the consistently documented involvement of parietal areas (specifically posterior parietal areas, including the intraparietal sulcus) in arithmetic and dyscalculia. This might also indicate that parietal deficits in dyscalculia may not be as consistent as has been claimed (see also Peters & De Smedt, in press).

Finally, the subject classification analysis differentiating children with dyslexia from children with dyscalculia did not yield any significant results, in any of the regions, for any of the formats. In line with the findings in the larger ROIs, children with different learning disorders cannot be distinguished by a trained classifier based on their neural activation patterns.

Subject generalizations in the smaller ROIs showed that generalization between groups of children with learning disorders based on neural activation patterns was possible for all formats in all regions under study. The only exceptions were the inferior frontal gyrus, where the generalization accuracy between children with dyslexia and children with comorbid dyslexia/dyscalculia just failed to reach significance for dots, and the fusiform gyrus, where generalization accuracies between children with dyslexia and children with dyscalculia and between children with dyslexia and children with comorbid dyslexia/dyscalculia did not reach significance for words.


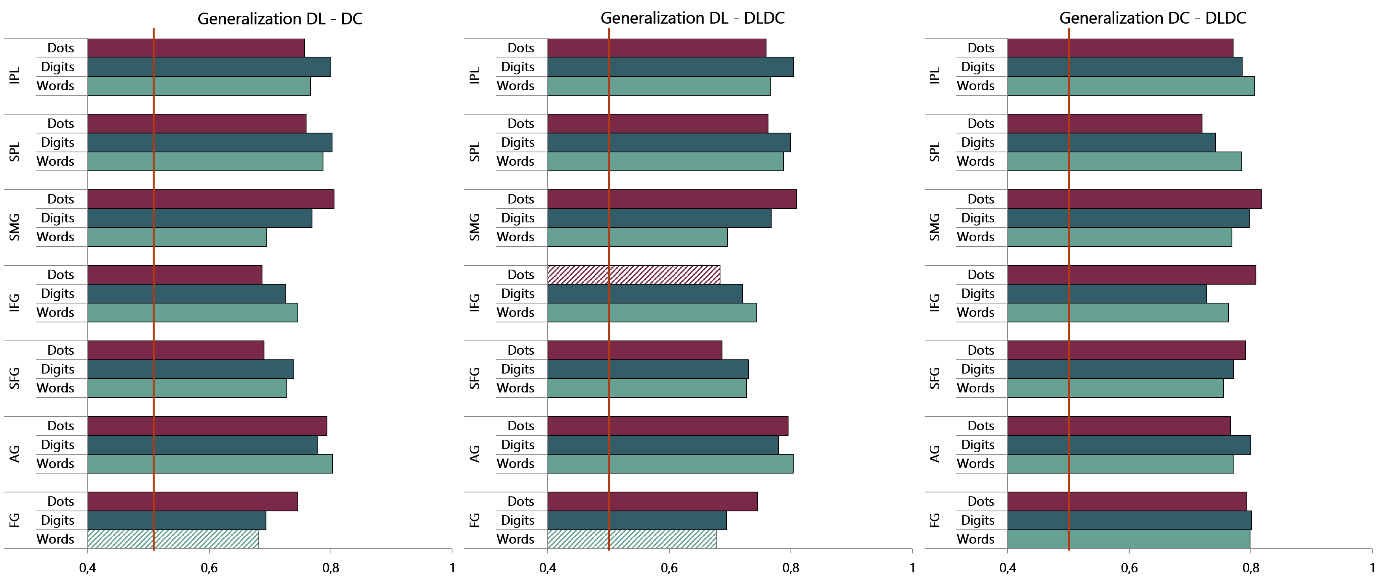


*Figure A.*2. Generalization accuracies per format (dots, digits and words) and per smaller ROI. Accuracies that reached significance are solidly filled, and chance level (0.50) is indicated.

**A.1 References**

Menon, V. (2015). Arithmetic in the child and adult brain. In R. Cohen Kadosh & A. Dowker (Eds.), *The Oxford Handbook Of Numerical Cognition*. Oxford University Press.

Peters, L., & De Smedt, B. (n.d.). Arithmetic in the developing brain: A review of brain imaging studies. *Developmental Cognitive Neuroscience*.
