# Supplementary material for: Dyscalculia and dyslexia: Different behavioral, yet similar brain activity profiles during arithmetic
Source: Neuroimage Clin. 2018 Mar 4;18:663–74. doi: 10.1016/j.nicl.2018.03.003 (PMC5987869; doi:10.1016/j.nicl.2018.03.003)
Supplement: Appendix B — Reading task. [file mmc2.docx]

**Appendix B: Reading task**

**B.1 Materials and methods**

Children also performed a reading task in the scanner, that was comparable to tasks used in previous fMRI studies on reading (e.g., Cao, Bitan, Chou, Burman, & Booth, 2006; Hoeft, Hernandez, Mcmillon, et al., 2006) adapted to Dutch. Because Dutch, just like French, has a more transparent orthography compared to English, the design of this task was based on the paradigm used in Simon, Mangin, Cohen, Le Bihan & Dehaene (2002). Children were visually presented with existing Dutch words, and were instructed to indicate either whether the word included the phoneme /e:/ (phoneme condition), or whether a word was presented in upper or lower case (visual condition). All words comprised the grapheme ‘e’, because in Dutch this grapheme has the most inconsistent grapheme-phoneme associations. For example, the grapheme ‘e’ can be read as /e:/ (as in ‘lego’ or ‘feest’ – *party*), as /i/ (as in ‘lied’ – *song*), as /ø/ (as in ‘neus’ – *nose*), as /ɛɪ/ (as in ‘reis’ – *journey*), as /u/ (as in ‘hoek’ – *corner*) or as /ə/ (as in ‘beton’ – *concrete*).

Fixation blocks and three task blocks were alternated and lasted 15 seconds each. A task block comprised a schematic instruction of the task at hand (1500ms), and four trials consisting of a short fixation (200ms) and the stimulus (3175ms). The schematic instruction consisted of a white face on a black background with an arrow pointed towards the ear for the phoneme condition (as children were instructed to check whether the grapheme ‘e’ *sounded like* /e:/), and with an arrow pointed towards the eye for the visual condition (as children had to *look* whether the letters were presented in upper case or in lower case). A run consisted of six blocks of both conditions (phoneme and visual) and lasted 255 seconds. Participants performed two runs. Words were presented in a white circle on a black background created by the Matlab script (Dehaene, Izard, & Piazza, 2005). Children were asked to press the left response button when an /e:/ sound was detected in the word, and the right button when the ‘e’ was associated with another phoneme for the phoneme condition. In the visual condition, children pressed the left response button when the word was presented in upper case, and the right response button when a word was presented in lower case. The design of the task is illustrated in Figure B.1. fMRI data were collected during two runs of the reading task at the end of the scanning sequence.


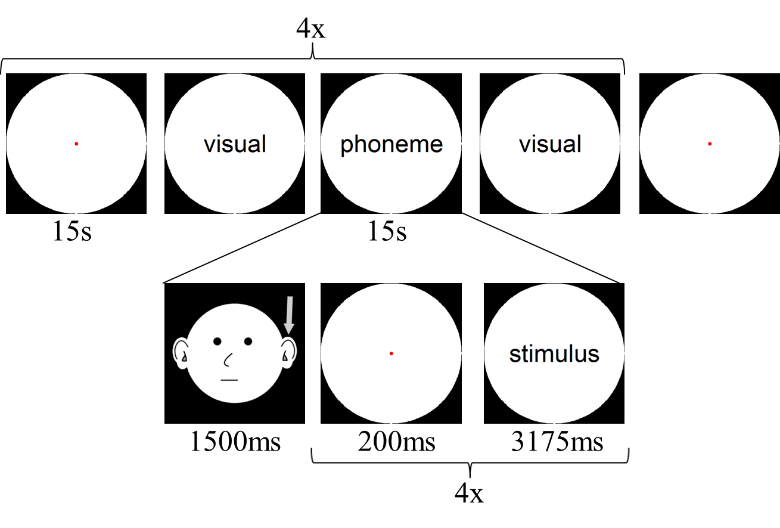


*Figure B.1.* Schematic overview of the reading task.

By definition, children with dyslexia and children with comorbid dyslexia/dyscalculia, but not children with only dyscalculia, were expected to perform more poorly on this task, and to show aberrant neural activation compared to their typically developing peers.

In the reading task, 14 participants were discarded due to excessive motion (2 TD, 6 DL, 4 DC and 2 DLDC), leading to a final sample of 45 children (21 TD, 11 DL, 7 DC and 6 DLDC). Of these remaining subjects, 13.33% of the runs of the reading task were discarded in the analyses due to motion. The four groups of children did not differ in degree of motion after this correction (*F*(3,41) = 1.07, *p* = .37). Due to the large loss of data – this task was presented at the end of the scanning sequence – these results should be interpreted with caution.

We calculated the effect size of 0.79 for the contrast “Dots-Fixation” for typically developing children compared to children with dyslexia (DL+DLDC) in the arithmetic task, which corresponds to a power of 0.82. In the reading task however, that the power (assuming an equal effect size for the reading and the arithmetic task) drops to 0.77 for TD vs. DL+DLDC contrasts, and even to 0.47 for contrasts between DL and DC.

**B.2 Results**

B.2.1 Behavioral results

Three-way ANOVAs with the presence of dyscalculia and the presence of dyslexia as between-subject factors, and condition (phoneme vs. visual) as within-subject factor were performed on the accuracy scores, the reaction times and the percentages non-response (see Figure B.2). Details on main and interaction effects per analysis are shown in Table B.1.


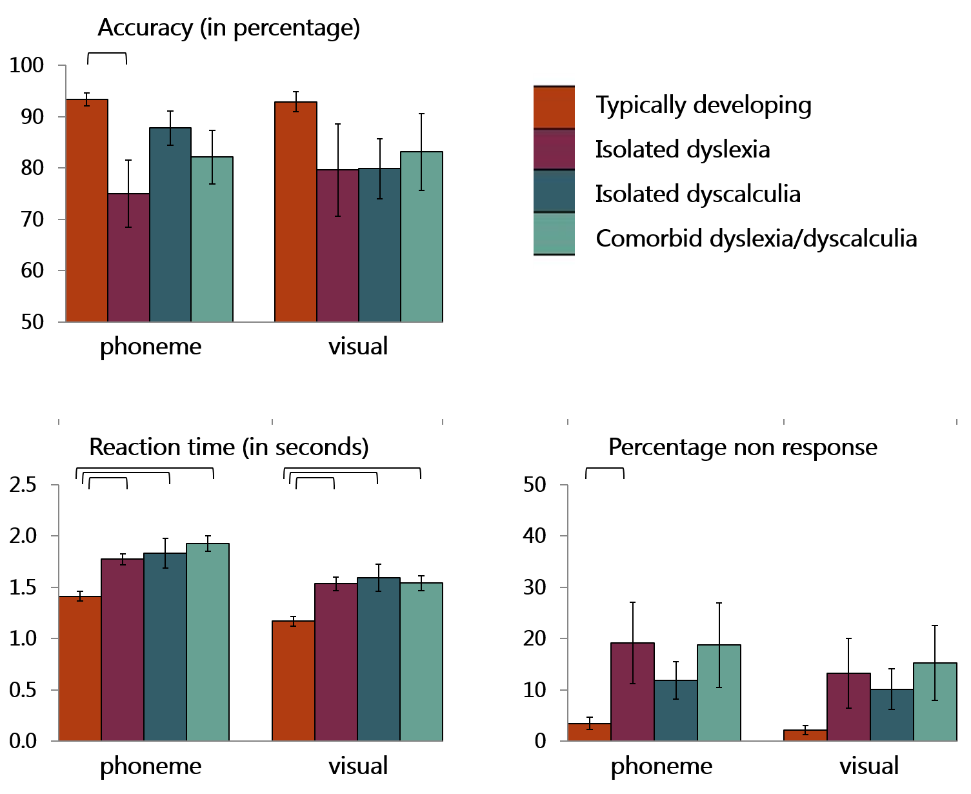


*Figure B.2.* Mean accuracy, reaction time (in seconds) and percentage non-response on the reading task per condition (phoneme on left, visual on right) per group. Error bars represent the standard error of the mean. Means connected by brackets differ significantly on a *p* < .05 level.

For the accuracy scores, there was no effect of condition (*F*(1,41) = .056, *p* = .82), nor of dyscalculia (*F*(1,41) = .21, *p* = .65). The main effect of dyslexia was marginally significant (*F*(1,41) = 3.82, *p* = .058), indicating a trend towards a lower performance for children with (DL+DLDC) compared to children without dyslexia (TD+DC). The interaction effect between dyslexia and dyscalculia was not significant (*F*(1,41) = 2.81, *p* = .10), indicating that arithmetic ability did not influence the fact that children with dyslexia (DL+DLDC) seemed to perform slightly worse than children without dyslexia (TD+DC). None of the other interaction effects reached significance (all *p*s > .25).

The analysis of the reaction times showed a main effect of condition (*F*(1,41) = 68.72, *p* < .001), with faster reaction times for the visual compared to the phoneme condition. Furthermore, both the main effect of dyslexia (*F*(1,41) = 7.31, *p* = .01) and dyscalculia (*F*(1,41) = 12.35, *p* = .001) were significant, indicating that children with dyslexia (DL+DLDC) and with dyscalculia (DC+DLDC) responded slower than children without dyslexia (TD+DC) and without dyscalculia (TD+DL), respectively. The interaction effect between dyslexia and dyscalculia (*F*(1,41) = 5.69, *p* = .022) reflected the fact that typically developing children responded faster than children with learning disorders (DL, DC and DLDC). None of the other effects reached significance (all *p*s > .26).

Finally, the analysis of the percentage non-response revealed a main effect of condition (*F*(1,41) = 7.15, *p* = .011) and of dyslexia (*F*(1,41) = 4.10, *p* = .05). Fewer responses were given in the phoneme condition than in the visual condition, and children with dyslexia (DL+DLDC) were more often late in responding than children without dyslexia (TD+DC). The interaction effect between dyslexia and dyscalculia was not significant (*F*(1,41) = 0.59, *p* = .45), indicating that arithmetic ability did not influence the main effect of dyslexia. All other effects did not reach significance (all *p*s > .19).

In summary, results showed that children with dyslexia (DL+DLDC) were less accurate and more often late in responding compared to typically developing children. As was the case in the arithmetic task, typically developing children responded faster than children with dyslexia, dyscalculia and comorbid dyslexia/dyscalculia.

Table B.1

*Main effects and interaction effects of the reading task*

|  |  | Accuracy | | Reaction time | | Non response | |
| --- | --- | --- | --- | --- | --- | --- | --- |
|  | *df* | *F* | *p* | *F* | *p* | *F* | *p* |
| Condition | 1,41 | 0.06 | .816 | 68.72 | < .001 | 7.15 | .011 |
| DL | 1,41 | 3.82 | .058 | 7.31 | .010 | 4.10 | .050 |
| DC | 1,41 | 0.21 | .646 | 12.35 | .001 | 0.89 | .352 |
| Condition x DL | 1,41 | 1.38 | .247 | 1.27 | .266 | 1.82 | .185 |
| Condition x DC | 1,41 | 0.85 | .361 | 1.13 | .294 | 0.17 | .684 |
| DL x DC | 1,41 | 2.81 | .101 | 5.69 | .022 | 0.59 | .446 |
| Condition x DL x DC | 1,41 | 0.11 | .746 | 1.31 | .259 | 0.39 | .537 |

B.2.2 Imaging results

*B.2.2.1 Univariate analyses*

The whole brain, full factorial ANOVAs performed on the two conditions of the reading task (phoneme and visual) showed no regions that were more active for one group of children compared to another at an FDR-corrected level (*p* < .05). At the uncorrected level (*p* < .001), typically developing children elicited more activation compared to children with dyslexia (DL+DLDC) and dyscalculia (DC+DLDC) in left superior parietal areas, left supramarginal gyrus, right cuneus, left superior temporal gyrus, and left medial frontal gyrus for the phoneme condition, and in left postcentral gyrus for the visual condition. Furthermore, based on visual inspection, the differences in activation appeared anatomically similar for typically developing children vs. children with dyslexia (DL+DLDC) compared to typically developing children vs. children with dyscalculia (DC+DLDC) for both conditions (see Figure B.3).

We would like to emphasize that these results were not driven by the inclusion of children from the comorbid group (DLDC) in all contrasts. As can be seen in Figure B.3, the group of children with isolated dyslexia (DL), which contains the largest number of children of any group of children with learning disorders (DL, DC and DLDC) showed the strongest effect.

Over both conditions, there were no brain regions that were recruited more by children with a learning disorder compared to typically developing children, also not at an uncorrected level (*p* < .001). Likewise, direct comparisons of children with learning disorders (DL vs. DC vs. DLDC) revealed no brain regions activated more by children from one group compared to another, also not at an uncorrected level (*p* < .001). Note that these analyses might lack power due to the lower number of participants in this second experiment.

Interestingly, we did not find differences in neural activation between the two conditions (phoneme vs. visual), for any of the subject groups, also not at the uncorrected level (*p* < .001). We suspect that this was the case because children also automatically read the words in the visual condition, as all stimuli were short, highly frequent Dutch words. A visual condition with scrambled letters would have been a task that might elicit more (albeit less interpretable) activation differences.


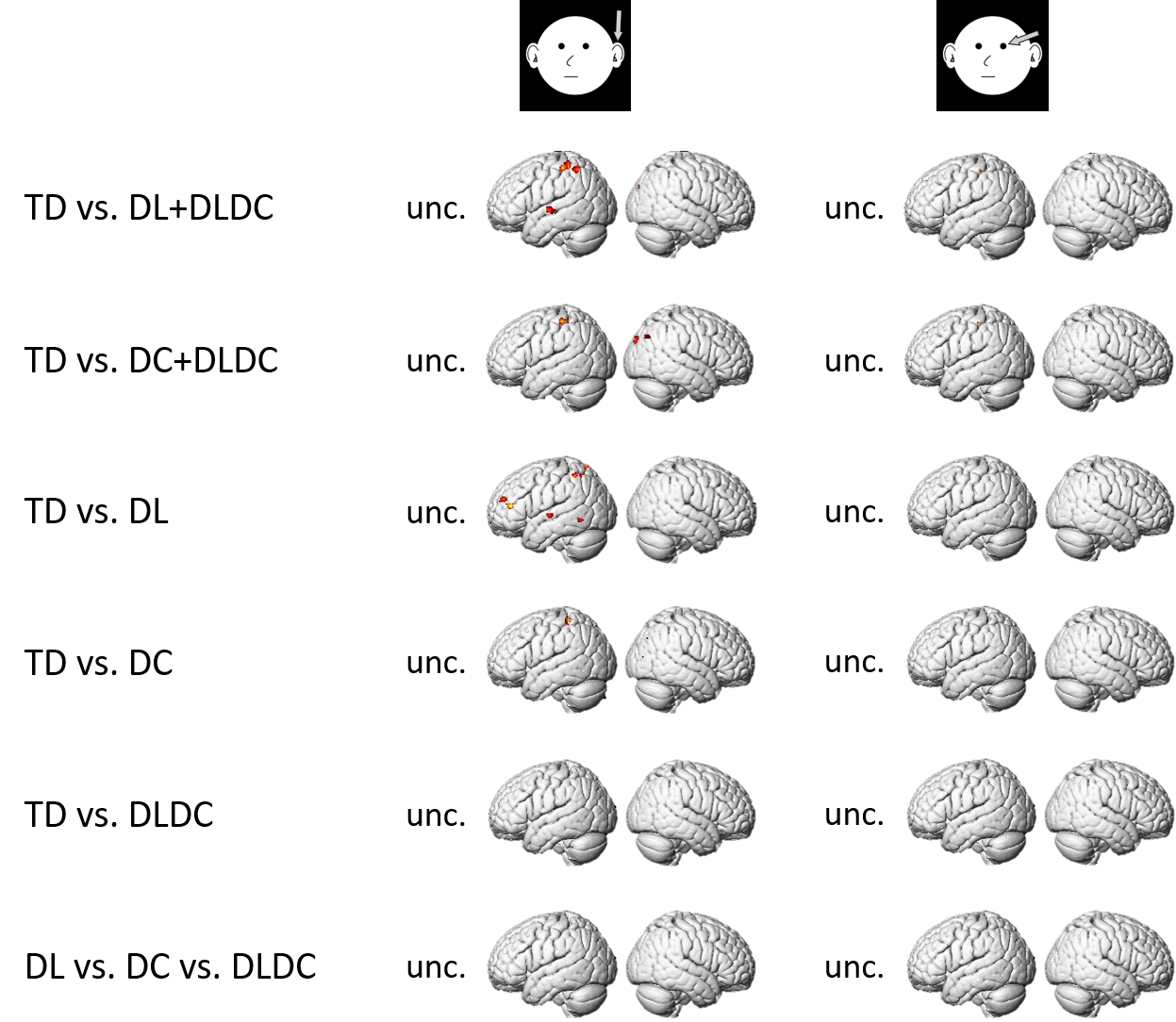


*Figure B.3.* Activation patterns of both conditions (phoneme, left and visual, right) of the reading task versus fixation, of TD vs. DL+DLDC, TD vs. DC+DLDC, TD vs. DL, TD vs. DC, TD vs. DLDC and DL vs. DC vs. DLDC. Activation patterns are shown uncorrected, because no activation clusters survived FDR correction.

*B.2.2.2 Subject classification analyses*

Subject classification analyses (see Figure B.4) were performed in all large ROIs to look into the (dis)similarity of neural activation patterns between groups. Classifying children as typically developing vs. having dyslexia was possible for both conditions at the whole brain level and in the frontal lobe. In the parietal lobe, only the activation patterns elicited by the phoneme condition allowed us to classify children with significant accuracies. In temporal and occipital lobe, classification accuracies did not reach significance for any of the conditions. Classifying children as typically developing vs. having dyscalculia on the other hand was possible for both conditions in the parietal lobe, and for the phoneme condition in occipital lobe. In the other large ROIs, classification did not reach significance. These results point towards different neural networks recruited for typically developing children compared to children with dyslexia or dyscalculia, although we cannot exclude the possibility that some of the apparent differences are related to a threshold effect and a low number of participants. We lack the power, for example, to claim that the nonsignificant classification of TD vs DC+ in the whole-brain ROI is significantly different from the significant classification of TD vs DL+ in that same ROI. This problem however, was overcome by the generalization analysis, which is documented in the next section of this appendix.


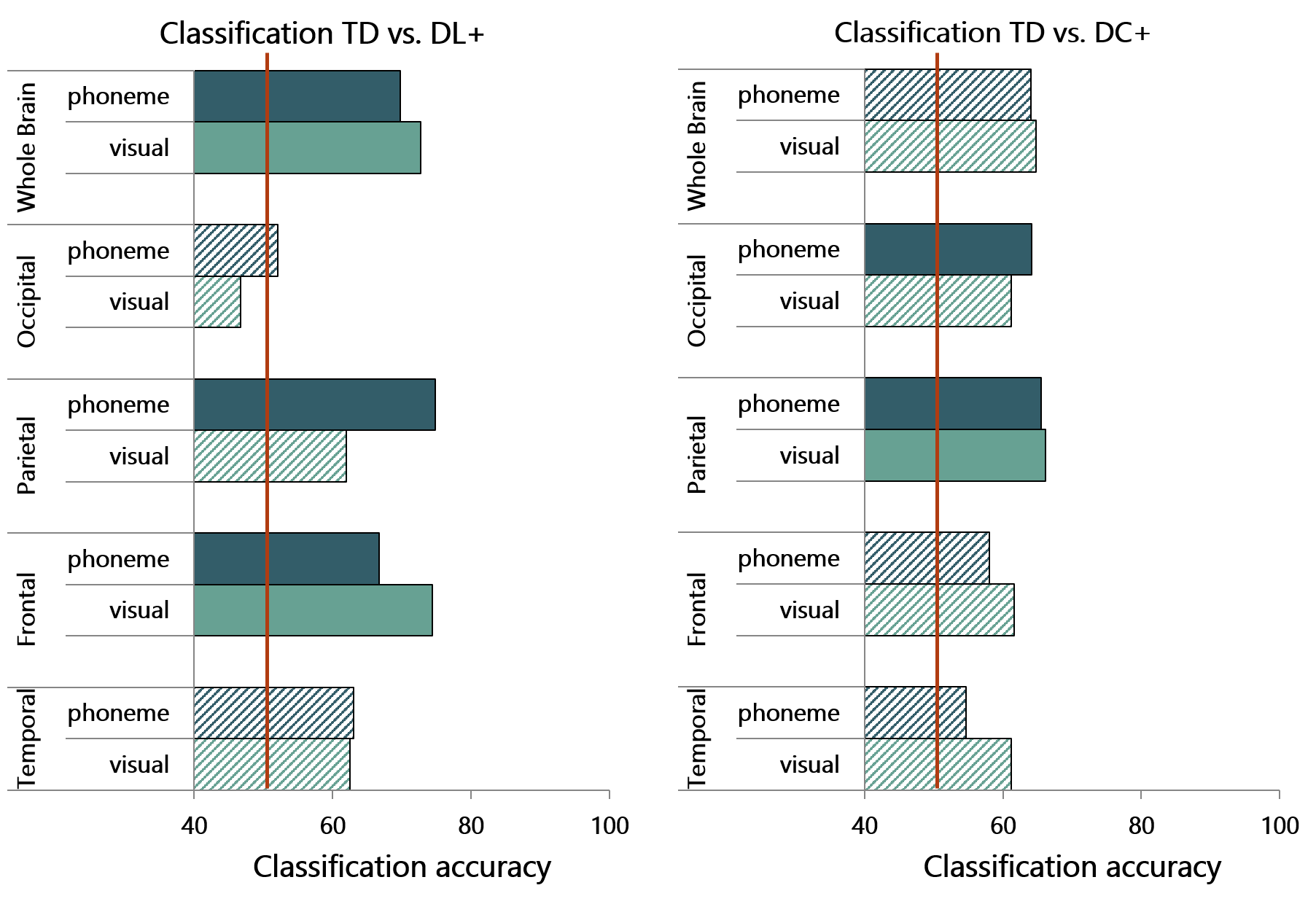


*Figure B.4* Classification accuracies per task (phoneme and visual) and per large ROI (whole brain, occipital, parietal, frontal and temporal lobes) for the reading task. Accuracies that reached significance are solidly filled in black or white, and chance level (0.50) is indicated.

*B.2.2.3 Subject generalization analyses*

Similarly compared to in the arithmetic task, visual inspection of the univariate results of the reading task showed that similar neural regions were activated less in children with dyslexia and dyscalculia compared to typically developing children (see Figure B.3), although caution is needed when interpreting these activation maps plotted at an uncorrected threshold. The data in the classification analyses revealed that it was not always the same ROI and the same task condition which showed significant classification effects, but again this finding is difficult to interpret given that many results were just below or above the statistical threshold. Furthermore, as argued above, such differences can only be meaningfully interpreted if pitted against the degree of similarity between the learning disorders under study. We therefore performed the same multi-voxel subject generalization analyses as in the arithmetic task on the reading task in all large ROIs to investigate whether the neural activation patterns of children with learning disorders were interchangeable, and hence were similar (see Figure B.5).

Activation patterns for children with dyslexia-only and children with dyscalculia-only were generalizable for both conditions on a whole brain level, and in parietal and frontal lobes. In the temporal lobe, generalization was only possible for the activation patterns elicited by the phoneme condition. In the occipital lobe, activation patterns were not similar enough to enable generalization. Activation patterns for children with dyslexia and children with comorbid dyslexia/dyscalculia were sufficiently similar to allow significant generalization on a whole brain level and in parietal and frontal lobes, for both conditions. In the occipital and temporal lobes, generalization was not possible for either condition. Finally, activation patterns of children with dyscalculia and children with comorbid dyslexia/dyscalculia allowed significant generalization for both conditions in parietal lobe and on a whole brain level, for the phoneme condition in occipital lobe, and for the visual condition in frontal lobe. In the temporal lobe, generalization between children with dyscalculia and children with comorbid dyslexia/dyscalculia did not reach significance. Despite a lack of power in this experiment, these results appear to be in line with the results obtained from the arithmetic task. This indicates that the neural activation patterns of different groups of children with learning disorders are remarkably similar, and distinct from the neural activation patterns of typically developing children.


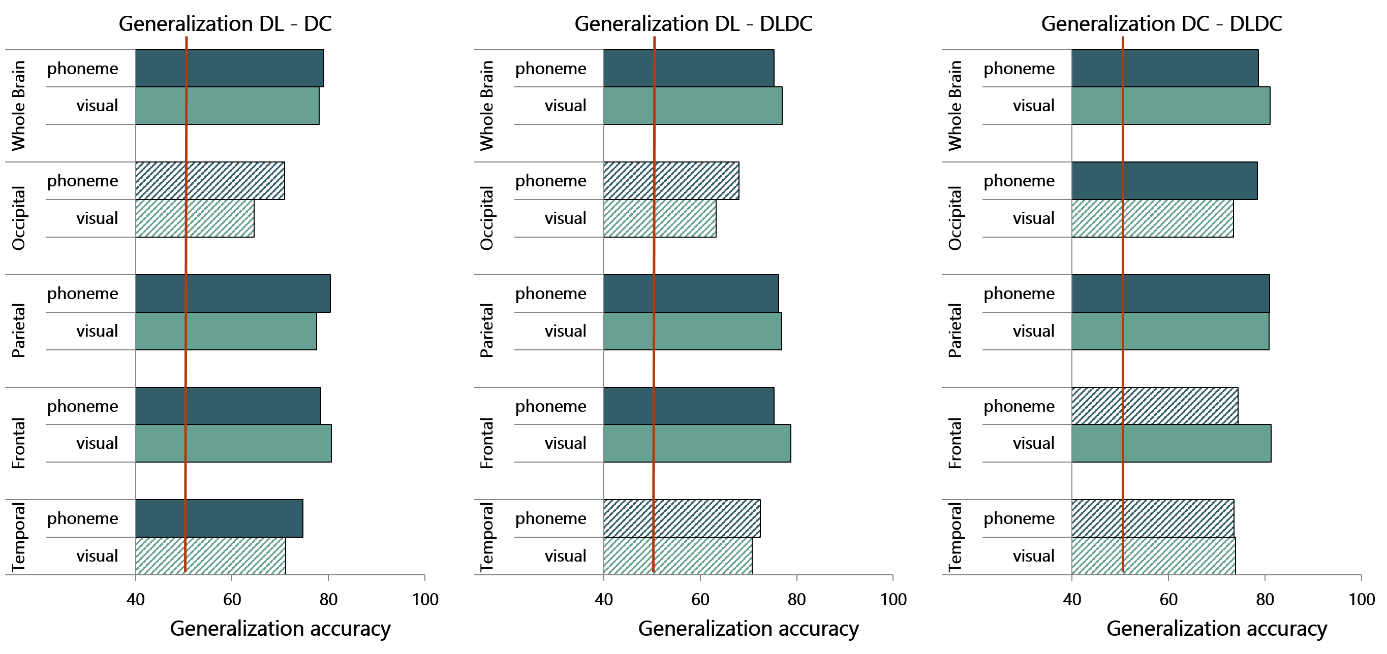


*Figure B.5.* Generalization accuracies per task (phoneme and visual) and per large ROI (whole brain, occipital, parietal, frontal and temporal lobes) for the reading task. Accuracies that reached significance are solidly filled in black or white, and chance level (0.50) is indicated.

**B.3 References**

Cao, F., Bitan, T., Chou, T. L., Burman, D. D., & Booth, J. R. (2006). Deficient orthographic and phonological representations in children with dyslexia revealed by brain activation patterns. *Journal of Child Psychology and Psychiatry and Allied Disciplines*, *47*, 1041–1050. doi:10.1111/j.1469-7610.2006.01684.x

Dehaene, S., Izard, V., & Piazza, M. (2005). Control over non-numerical parameters in numerosity experiments. Unpublished manuscript (available at www.unicog.org).

Hoeft, F., Hernandez, A., Mcmillon, G., Taylor-hill, H., Martindale, J. L., Meyler, A., … Gabrieli, J. D. E. (2006). Neural basis of dyslexia: A comparison between dyslexic and nondyslexic children equated for reading ability, *26*(42), 10700–10708. doi:10.1523/JNEUROSCI.4931-05.2006

Simon, O., Mangin, J. F., Cohen, L., Le Bihan, D., & Dehaene, S. (2002). Topographical layout of hand, eye, calculation, and language-related areas in the human parietal lobe. *Neuron*, *33*(44), 475–487. doi:10.1016/S0896-6273(02)00575-5
